# l-valine is a powerful stimulator of GLP-1 secretion in rodents and stimulates secretion through ATP-sensitive potassium channels and voltage-gated calcium channels

**DOI:** 10.1038/s41387-024-00303-4

**Published:** 2024-06-11

**Authors:** Ida Marie Modvig, Mark M. Smits, Katrine Douglas Galsgaard, Anna Pii Hjørne, Anna Katarzyna Drzazga, Mette Marie Rosenkilde, Jens Juul Holst

**Affiliations:** 1 Department of Biomedical Sciences, Faculty of Health and Medical Sciences, University of Copenhagen, Copenhagen, Denmark; 2 Institute of Molecular and Industrial Biotechnology, Faculty of Biotechnology and Food Sciences, Lodz University of Technology, Łódź, Poland; 3 Novo Nordisk Foundation Center for Basic Metabolic Research, University of Copenhagen, Copenhagen, Denmark

**Keywords:** Type 2 diabetes, Type 2 diabetes, Obesity

## Abstract

**Background:**

We previously reported that, among all the naturally occurring amino acids, l-valine is the most powerful luminal stimulator of glucagon-like peptide 1 (GLP-1) release from the upper part of the rat small intestine. This makes l-valine an interesting target for nutritional-based modulation of GLP-1 secretion. However, the molecular mechanism of l-valine-induced secretion remains unknown.

**Methods:**

We aimed to investigate the effect of orally given l-valine in mice and to identify the molecular details of l-valine stimulated GLP-1 release using the isolated perfused rat small intestine and GLUTag cells. In addition, the effect of l-valine on hormone secretion from the distal intestine was investigated using a perfused rat colon.

**Results:**

Orally given l-valine (1 g/kg) increased plasma levels of active GLP-1 comparably to orally given glucose (2 g/kg) in male mice, supporting that l-valine is a powerful stimulator of GLP-1 release in vivo (*P* > 0.05). Luminal l-valine (50 mM) strongly stimulated GLP-1 release from the perfused rat small intestine (*P* < 0.0001), and inhibition of voltage-gated Ca^2+^-channels with nifedipine (10 μM) inhibited the GLP-1 response (*P* < 0.01). Depletion of luminal Na^+^ did not affect l-valine-induced GLP-1 secretion (*P* > 0.05), suggesting that co-transport of l-valine and Na^+^ is not important for the depolarization necessary to activate the voltage-gated Ca^2+^-channels. Administration of the K_ATP_-channel opener diazoxide (250 μM) completely blocked the l-valine induced GLP-1 response (*P* < 0.05), suggesting that l-valine induced depolarization arises from metabolism and opening of K_ATP_-channels. Similar to the perfused rat small intestine, l-valine tended to stimulate peptide tyrosine-tyrosine (PYY) and GLP-1 release from the perfused rat colon.

**Conclusions:**

l-valine is a powerful stimulator of GLP-1 release in rodents. We propose that intracellular metabolism of l-valine leading to closure of K_ATP_-channels and opening of voltage-gated Ca^2+^-channels are involved in l-valine induced GLP-1 secretion.

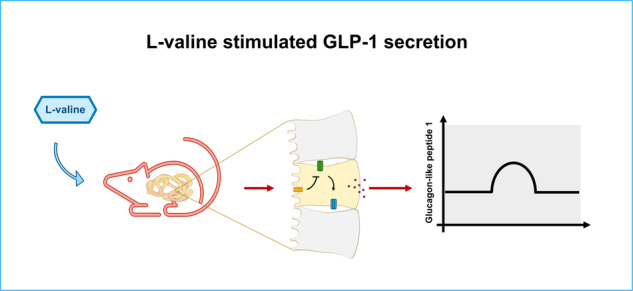

## Introduction

Glucagon-like peptide 1 (GLP-1) is an important regulator of glucose homeostasis and a key factor in the regulation of energy intake [1–5]. It has received much attention due to the successful application of GLP-1 receptor agonists (GLP-1RAs) in the treatment of both type 2 diabetes (T2D) and obesity [6]. GLP-1 is secreted from enteroendocrine cells in the gastrointestinal tract in response to ingestion of nutrients [1, 7–9]. Targeting the endogenous secretion of GLP-1 by nutritional modulation may be a valuable alternative to existing GLP-1RAs, as the appetite- and blood glucose-lowering effects of GLP-1 are thought to be, at least partly, caused by activation of local sensory nerves (immediately after its release) [10, 11], which may not be engaged by GLP-1RAs. Additionally, targeting GLP-1 secretion may also lead to increased secretion of other gut hormones, as the L-cells produce and secrete multiple hormones [12–16], which likewise are insulinotropic (oxyntomodulin) and/or act to inhibit appetite (peptide tyrosine-tyrosine; PYY), thereby enhancing the insulinotropic and appetite reducing effect [13]. Finally, as targeting the endogenous secretion would reflect normal physiology more closely, this may not be associated with the side effects experienced by some patients receiving GLP-1RAs.

However, our knowledge of the regulation of endogenous GLP-1 secretion is far from exhaustive. In particular, the mechanisms by which products from protein digestion stimulate GLP-1 release remain unclear, as digestion of proteins generates a mixture of oligopeptides and various amino acids, making the study of absorption and sensory mechanisms challenging, as sensing of these degradation products might involve several molecular triggers and stimulatory pathways.

Previously, we identified potent stimulation of GLP-1 secretion from isolated perfused rat intestinal preparations with protein hydrolysates [17], and we demonstrated that the stimulation was associated with absorption [17]. Since absorbed protein digestion products are thought to be degraded intracellularly to individual amino acids, we hypothesized that the amino acids are generally responsible for the GLP-1 stimulatory effect of dietary proteins. When tested in perfusion setups, we observed that l-valine was distinctively the most powerful luminal stimulator of GLP-1 release from the upper part of the rat small intestine [18] raising the possibility that dietary supplementation with l-valine may serve as a potential strategy to increase endogenous GLP-1 secretion.

Therefore, in this study, we wished to investigate the molecular details of l-valine’s stimulatory effect on gut hormone secretion. As l-valine (in previous studies [18]) only stimulated GLP-1 secretion when infused from the luminal side of the intestine, we hypothesized that the mechanism of GLP-1 stimulation was coupled to the uptake and/or intracellular mechanisms. Therefore, we investigated whether the response depends on co-transport of sodium and membrane depolarization; whether it depends on amino acid oxidation; or whether calcium influx is involved.

## Results

### Orally given l-valine stimulates GLP-1 secretion similar to oral glucose in mice

**Fig. 1:**
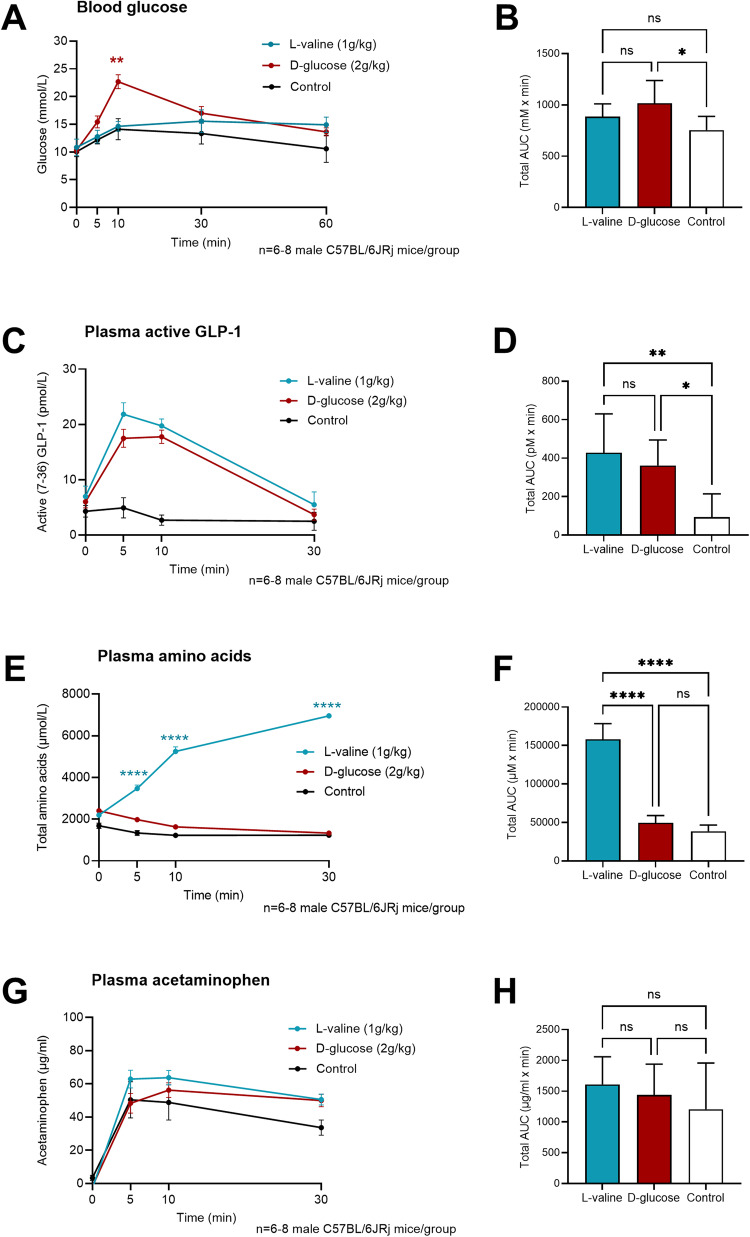
Effect of orally given l-valine on blood glucose and GLP-1 secretion in male mice. **A** Blood glucose concentration (mmol/L) shown as means ± SEM, *n* = 8 male C57BL/6JRj mice in l-valine group and d-glucose group and *n* = 6 male C57BL/6JRj mice in control group (water). Blue line: Oral l-valine (1 g/kg). Red line: Oral d-glucose (2 g/kg). Black line: Control group (water). **P < 0.01 at timepoint 10 min compared to l-valine and control (water) group. **B** Total areas under the curves (AUCs) of blood glucose (mmol/L x min). *P < 0.05. **C** Plasma concentration of active (7–36) GLP-1 (pmol/L) shown as means ± SEM. **D** Total areas under the curves (AUCs) of plasma GLP-1 (pmol/L x min). *P < 0.05, **P < 0.01. **E** Total amino acid concentration in plasma (µmol/L) shown as means ± SEM. ****P < 0.0001 at timepoint 5, 10, and 30 min compared to d-glucose and control group. **F** Total areas under the curves (AUCs) of total amino acids in plasma (µmol/L × min). ****P < 0.0001. **G** Plasma concentration of acetaminophen (µg/ml) shown as means ± SEM. **H** Total areas under the curves (AUCs) of plasma acetaminophen in plasma (µg/ml x min). NS, nonsignificant, *P* > 0.05.

To test the effect of l-valine in vivo, male C57BL/6JRj mice (*n* = 8/group) received either l-valine (1 g/kg), d-glucose (2 g/kg) or water (control group, *n* = 6) orally. At timepoint −30 min, mice received an oral cocktail consisting of the neprilysin (NEP) inhibitor sacubitril (5 uL/g) and the dipeptidyl peptidase (DPP)-4 inhibitor sitagliptin (5 uL/g) based on previous findings on how to optimize the measurement of active GLP-1 in mice [19]. Blood samples were collected at time points 0, 5, 10, and 30 min. Blood glucose concentrations were similar across groups at baseline (min 0; *P* > 0.99). At time point 10 min, peak blood glucose levels differed significantly between mice receiving glucose and mice receiving l-valine or water (*P* < 0.002) (Fig. 1A) but the total areas under the curves (tAUC0-60 min) did not differ between the l-valine group and the d-glucose group (*P* > 0.05) (Fig. 1B).

Active GLP-1 (7–36) levels increased in both the l-valine and the d-glucose treated group with a peak at timepoint 5 min for the l-valine group (21.84 ± 5.9 pM) and 10 min for the d-glucose group (17.76 ± 3.4 pM; Fig. 1C). The explanation for this could be differences in absorption kinetics as well as differences in the molecular pathways underlying l-valine and d-glucose stimulated GLP-1 secretion. The total areas under the curves (tAUC0-60 min), however, did not differ between the l-valine and the d-glucose group (*P* > 0.05; Fig. 1D), suggesting that l-valine and d-glucose are equipotent with respect to GLP-1 release in vivo in mice. We also measured the absorption of l-valine reflected by an increase in plasma total amino acids to validate that l-valine was being absorbed within the time frame of the GLP-1 peak. The plasma amino acid concentration increased significantly at timepoint 5 min (*P* < 0.0001) compared to the d-glucose and the control group, and continued increasing throughout the experimental protocol, supporting that l-valine was rapidly absorbed (within the first 5 min; Fig. 1E, F).

Gastric emptying, reflected by changes in plasma acetaminophen concentrations did not differ between l-valine treated, d-glucose treated, and water treated mice (*P* > 0.05) (Fig. 1G, H).

### l-valine stimulates GLP-1 secretion in perfused rat small intestines and GLUTag cells

**Fig. 2:**
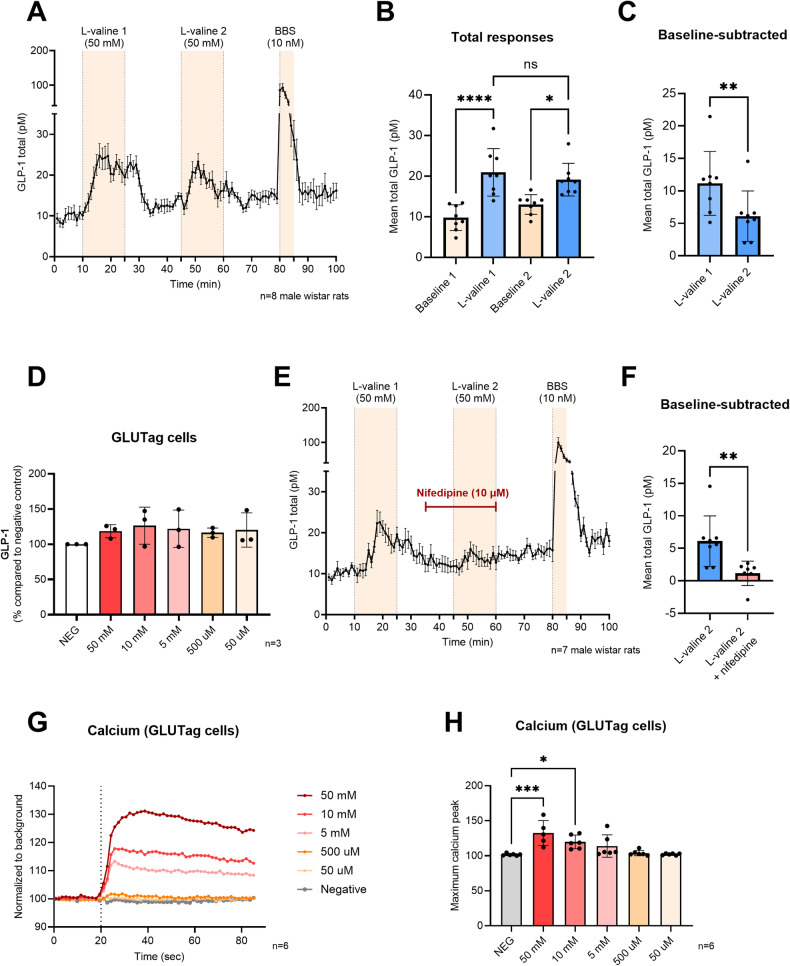
Activation of V-gated Ca^2+^-channels is involved in l-valine stimulated GLP-1 secretion. **A** Total GLP-1 secretion from the perfused rat small intestine (pmol/L ± SEM). l-valine was administered intra-luminal between minute 11 and 25 and between minute 46 and 60 at a concentration of 50 mmol/L. The positive control, bombesin (BBS), was administered intra-vascularly between minute 81 and 85, *n* = 8 male Wistar rats. **B** Mean total GLP-1 concentrations (pmol/L ± SD) at baseline (baseline 1 and 2) and during l-valine stimulation (l-valine 1 and 2). **C** Mean baseline-subtracted GLP-1 concentrations (pmol/L ± SD) in response to l-valine 1 and l-valine 2 stimulation. **D** GLP-1 secretion from GLUTag cells relative to negative control in response to different concentrations of l-valine. The negative control was set as 100%, and the different GLP-1 responses presented relative to that, *n* = 3 (including 3 technical replicates/experiment). **E** Total GLP-1 secretion from the perfused rat small intestine (pmol/L ± SEM). l-valine was administered intra-luminal between minute 11 and 25 and between minute 46 and 60. Nifedipine (inhibitor of V-gated Ca^2+^-channels; 10 µmol/L) was administered intra-vascularly between minute 36 and 60, *n* = 7 male Wistar rats. **F** Mean baseline-subtracted GLP-1 concentrations (pmol/L ± SD) in response to l-valine 2 (Fig. 2C) and l-valine 2 + nifedipine. **G** Real-time calcium fluorescence (using Fluo-4) in GLUTag cells in response to different l-valine concentrations. Data is normalized to background, *n* = 6 (including 2–3 technical replicates/experiment). **H** Maximum peaks of real-time calcium fluorescence in response to different l-valine concentrations, *n* = 6 (including 2–3 technical replicates/experiment). NS nonsignificant, *P* > 0.05. *P < 0.05, **P < 0.021, ***P < 0.0002, ****P < 0.0001.

In isolated perfused small intestines (*n* = 8) from Wistar rats, luminal infusion of l-valine (50 mM) powerfully stimulated GLP-1 secretion in a control experiment with repeated stimulation (*p* < 0.0001), in agreement with previous observations [18] (Fig. 2A). These experiments served as controls to be compared with subsequent experiments. When comparing the total responses, there were no significant differences between the first and the second l-valine stimulation (20.9 ± 5.8 pM vs. 19.1 ± 4 pM, *P* > 0.05; Fig. 2B), however when comparing the baseline-subtracted responses, the second l-valine response was significantly lower than the first (11.2 ± 4.9 pM vs. 6.1 ± 3.9 pM, *P* < 0.002; Fig. 2C). Therefore, in subsequent experiments, the second l-valine response was compared to the second l-valine response in the control experiment. The positive control bombesin (BBS) markedly stimulated GLP-1 secretion in all experiments, indicating that the L-cells were fully responsive at the end of the protocol (Fig. 2A).

Next, we tested the GLP-1 secreting potential of different concentrations of l-valine using the in vitro GLUTag cell line (kindly provided by Professor Drucker from the University of Toronto in Canada). All of the tested concentrations (50 µM to 50 mM) numerically increased GLP-1 secretion (Fig. 2D).

### Voltage-gated calcium channels are involved in l-valine induced GLP-1 secretion

To test the involvement of voltage-gated calcium channels (V-gated Ca^2+^-channels) in l-valine induced GLP-1 secretion, the Ca^2+^-channel blocker nifedipine (10 µM) was infused vascularly in the perfused rat small intestine from 10 min before the second l-valine stimulation until the end of the stimulation period (minutes 36–60; Fig. 2E). The concentration and timing of nifedipine stimulation was based on control studies as well as previous studies in the perfused rat small intestine [17, 20], where nifedipine (10 µM) inhibited glucose-stimulated GLP-1 secretion, which has been demonstrated by several groups to be driven by opening of V-gated Ca^2+^-channels.

Stimulation intervals used for statistical analysis were minutes 47–56 (l-valine 2 + nifedipine and l-valine 2 in control experiment). In combination with nifedipine, the second stimulation was much reduced (l-valine 2 + nifedipine) compared to the second stimulation in the control experiment (l-valine 2, Fig. 2C; 1.2 ± 1.9 pM vs. 6.1 ± 3.9 pM, *p* < 0.01, *n* = 7–8; Fig. 2F), indicating that nifedipine blocked l-valine induced GLP-1 secretion during the second stimulation.

We further assessed the calcium-dependency of l-valine in GLUTag cells using Fluo-4 calcium assay, which allows real-time detection of intracellular calcium mobilization. l-valine concentrations of 10 mM and 50 mM increased intracellular calcium levels significantly upon exposure (*P* < 0.05, *n* = 6; Fig. 2G, H).

### Sodium does not seem to be essential for l-valine induced GLP-1 secretion

**Fig. 3:**
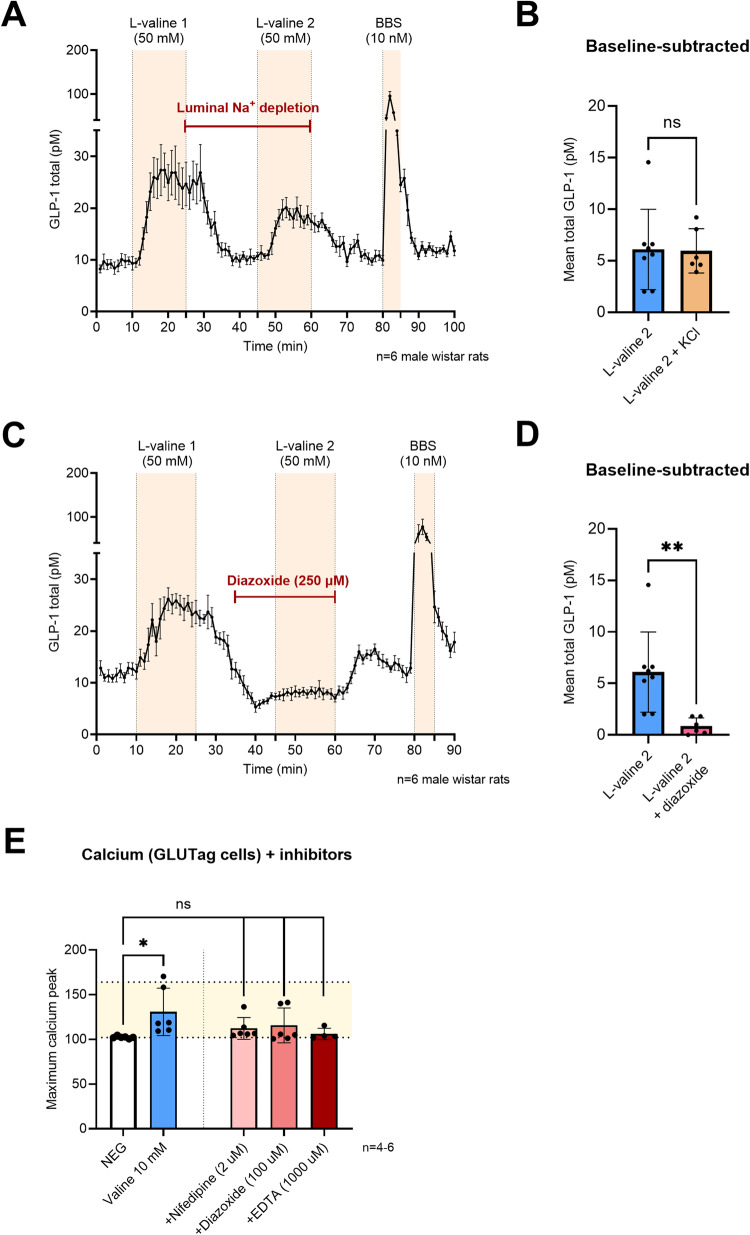
l-valine induced membrane depolarization arises from intracellular metabolism of l-valine and closure of K_ATP_-channels. **A** Total GLP-1 secretion from the perfused rat small intestine (pmol/L ± SEM). l-valine (50 mmol/L) was administered intra-luminal between minutes 11 and 25 and between minutes 46 and 60. From minute 26 to 60, luminal NaCl was replaced with KCl (0.11%), *n* = 6 male Wistar rats. **B** Mean baseline-subtracted GLP-1 concentrations (pmol/L ± SD) in response to l-valine 2 (Fig. 2C) and l-valine 2 + KCl. **C** Total GLP-1 secretion from the perfused rat small intestine (pmol/L ± SEM). l-valine was administered intra-luminal between minutes 11 and 25 and between minutes 46 and 60. Diazoxide (opener of K_ATP_-channels; 250 µmol/L) was administered intra-vascularly between minutes 36 and 60, *n* = 6 male Wistar rats. **D** Mean baseline-subtracted GLP-1 concentrations (pmol/L ± SD) in response to l-valine 2 (Fig. 2C) and l-valine 2 + diazoxide. **P < 0.021. **E** Maximum peaks of real-time calcium fluorescence in response to l-valine, l-valine + nifedipine (2 µmol/L), diazoxide (100 µmol/L) and Ethylenediaminetetraacetic Acid Tetrasodium Salt (EDTA; 1 mmol/L), *n* = 4–6 (including 2–3 technical replicates/experiment). NS, nonsignificant, *P* > 0.05. *P < 0.05.

Next, we wanted to examine if Na^+^-coupled uptake was causing l-valine induced membrane depolarization in the intestinal GLP-1 secreting cells, as L-amino acids can enter the cell via Na^+^-coupled transport. l-valine (50 mM) was infused luminally at two time points (minutes 11–25 and 46–60), separated by a 20-minute washing period. During the washing period and the second stimulation, the luminal saline solution was replaced with an isosmotic solution of KCl (0.11%) to deprive the perfused intestine of luminal Na^+^ (Fig. 3A). Stimulation intervals used for statistical analyses were minutes 47–56 (l-valine 2 + KCl). However, substitution with KCl did not influence baseline-subtracted GLP-1 responses to l-valine (6.0 ± 2.1 pM vs. 6.1 ± 3.9 pM, *P* > 0.05, *n* = 6–8; Fig. 3B), indicating that Na^+^ entry is not necessary for l-valine induced membrane depolarization and GLP-1 secretion.

### Opening of ATP-sensitive potassium channels inhibits l-valine induced GLP-1 secretion

To investigate whether intracellular metabolism of l-valine leading to the production of ATP and closure of K_ATP_-channels, subsequently resulting in impaired K^+^ efflux and cellular depolarization, was involved in the mechanisms of l-valine stimulated GLP-1 secretion, we infused the K_ATP_-channel opener diazoxide (250 µM) while stimulating with l-valine (Fig. 3C). Diazoxide powerfully inhibited the l-valine induced GLP-1 response when comparing baseline-subtracted values (6.1 ± 3.9 pM vs. 0.9 ± 0.8 pM, *P* < 0.01, *n* = 6–8; Fig. 3D), supporting that the upstream pathway leading to membrane depolarization and opening of V-gated Ca^2+^-channels might be intracellular metabolism of l-valine followed by the closure of K_ATP_-channels.

Using Fluo-4 experiments in GLUTag cells, preincubation with diazoxide reduced the effect of 10 mM l-valine on intracellular calcium, making it no longer statistically significant (Fig. 3E). Pre-incubation of the cells with nifedipine (2 µM) or EDTA (1 mM), a calcium-chelating agent, likewise reduced the effect of 10 mM l-valine (Fig. 3E), suggesting that all these inhibitors affect the l-valine induced increase in intracellular calcium.

### l-valine is absorbed and increases GLP-1 and PYY secretion from the perfused rat colon

As hormone secretion induced by amino acids in the lower part of the intestine, where the majority of GLP-1 secreting cells are present [21], remained to be investigated, and as l-valine stimulated GLP-1 secretion in GLUTag cells, which is a colon-derived cell line, we further wished to examine the effect of l-valine on hormone secretion from the perfused rat colon.

**Fig. 4:**
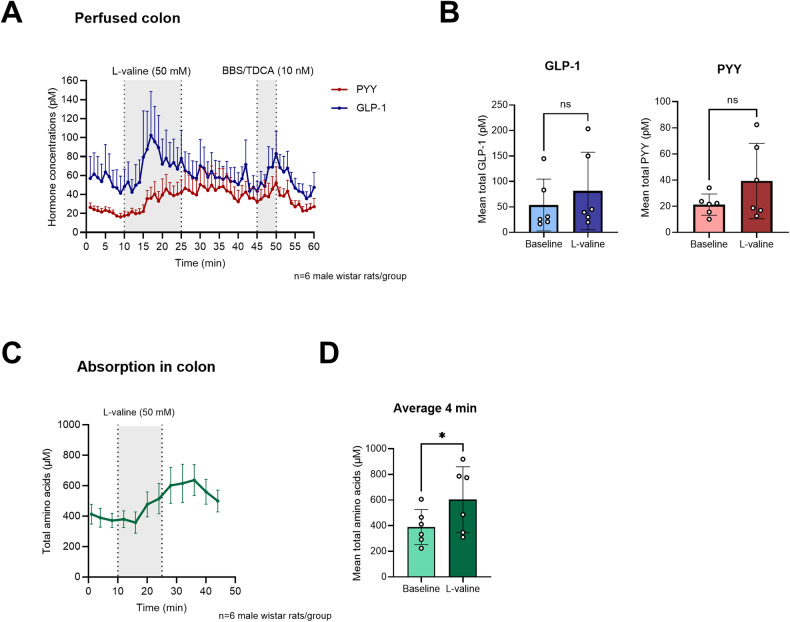
l-valine is absorbed and increases GLP-1 and PYY secretion from the perfused rat colon. **A** Total GLP-1 (blue) and PYY (red) secretion from the perfused rat colon (pmol/L ± SEM). l-valine (50 mmol/L) was administered intra-luminal between minute 11 and 25. The positive control, bombesin (BBS) mixed with the bile acid, Tauro deoxycholic acid (TDCA; 1 mM), was administered intra-vascularly between minute 46 and 50, *n* = 6 male Wistar rats. **B** Mean total GLP-1 (blue) and PYY (red) concentrations (pmol/L ± SD) at baseline and during l-valine stimulation. **C** Total amino acids in perfusion effluents of perfused colon in response to l-valine stimulation (µmol/L ± SEM), *n* = 6. **D** Mean total amino acid concentrations (µmol/L ± SD) at baseline and during l-valine stimulation. NS, nonsignificant, *P* > 0.05. *P < 0.05.

Luminal infusion of l-valine (50 mM) into the perfused rat colon increased both GLP-1 and PYY secretion although not significantly when compared to baseline secretion (GLP-1: 53.7 ± 20.8 pM vs. 81.4 ± 31.2 pM, *p* = 0.0575, PYY: 21.2 ± 3.3 pM vs. 39.3 ± 11.8 pM, *P* > 0.05, *n* = 6; Fig. 4A, B). To investigate whether the weaker hormone response (compared to the responses in the small intestine) was due to a decreased absorption of l-valine in the colon, we measured the absorption of l-valine. l-valine was indeed absorbed (baseline: 388.5 ± 55.8 µM vs. l-valine: 603.7 ± 104.4 µM, *P* < 0.05, *n* = 6), but not as effectively as in the perfused proximal rat small intestine (with increases from ~400 to 2000 µM) [18], supporting the theory that the stimulation of GLP-1 secretion by l-valine is coupled to absorption of the amino acid.

## Methods

### Animals and ethical considerations

Male Wistar rats ( ~ 250 g, 9 weeks) and male C57BL/6JRj mice (~25 g,12 weeks) were obtained from Janvier (Le Genest-Saint-Isle, France) and housed four rats per cage and eight mice/cage (allowing 102,5 cm^2^/mouse). Mice and rats were allowed one week of acclimatization and kept on a 12:12 h light/dark cycle with ad libitum access to water and standard chow.

Studies were conducted with permission from the Danish Animal Experiments Inspectorate (2023-15-0201-01408) and the local ethical committee (EMED P23-262 and P23-263) in accordance with the EU Directive 2010/63/EU and guidelines of Danish legislation governing animal experimentation (1987) and the National Institute of Health.

### In vivo studies

Experiments were carried out on two occasions on mice (26.3 ± 1.9 g) fasted for 5 h. Mice were divided into weight-matched groups, (*n* = 8; l-valine and d-glucose group, *n* = 6; control group). At −30 min, mice received a cocktail consisting of the neprilysin (NEP) inhibitor, sacubitril (0.3 mg/kg, 5 uL/g, cat. no. 333-B1070, Nordic Biosite, Täby, Sweden) and the dipeptidyl peptidase (DPP)-4 inhibitor, sitagliptin (10 mg/kg, 5 uL/g, Xelevia; Merck, Sharp & Dohme) orally as described elsewhere [19]. At time 0 min, 75 uL tail blood was collected into ethylenediaminetetraacetic acid (EDTA) coated capillary tubes (cat. no. 167313, Micro Haematocrit Tubes, Vitrex Medical A/S, Herlev, Denmark) and instantly transferred onto ice. At time 0 min, mice were given orally either l-valine (1 g/kg, 20 uL/g), d-glucose (2 g/kg, 20 uL/g) or milliQ water (20 uL/g) mixed with acetaminophen (100 mg/kg). l-valine and d-glucose were prepared in milliQ water. Mice from the same cage received different treatments. Tail blood was collected at times; 0, 5, 10 and 30 min. Glucose concentration was measured prior to collection of blood samples. An extra blood sample for glucose measurement was taken at time 60 min. After blood collection, the mice were euthanized by cervical dislocation. Blood samples were centrifuged (6500 rpm, 4 °C, 10 min) within half an hour to obtain plasma. Plasma was transferred to pre-chilled PCR tubes (Thermowell, Gold PCR; Corning, NY) and placed on ice until storage at −20 °C.

### Isolation and perfusion of the rat small and large intestine

Non-fasted rats were anaesthetized with a subcutaneous injection of Hypnorm/Midazolam (0.0158 mg fentanyl citrate+0.5 mg fluanisone+0.25 mg midazolam/100 g). When lack of reflexes was established, rats were placed on a heating plate (37 °C), and the abdominal cavity opened. The proximal small intestine (~37 cm) was isolated by ligating the vascular supply to the distal part and removing the colon. The colon was isolated by ligating the vascular supply to the cecum, the small intestine, the spleen, the stomach, the kidneys and the celiac artery, allowing isolation of the most proximal part of the colon to the part just proximal to the entry of inferior mesenteric artery (~10 cm). A plastic tube was placed in the lumen of the proximal small/large intestine, and the intestine was gently flushed with isotonic saline (room temperature) to remove luminal contents. Throughout the experimental protocol, a constant luminal flow of saline was applied via a syringe pump (0.25 ml/min; small intestine, 0.15 ml/min; colon). For perfusion of the small intestine, a catheter was inserted into the superior mesenteric artery, and the intestine was vascularly perfused with heated (37 °C), oxygenated (95% O_2_ and 5% CO_2_) perfusion buffer at a constant flow rate of 7.5 ml/min using a single pass perfusion system (UP100, Hugo Sachs Harvard Apparatus, Germany). For perfusion of the colon, a catheter was inserted into the abdominal aorta, which was ligated proximally to the superior mesenteric artery, tributaries to the small intestine were ligated and the colon was vascularly perfused at a constant flow rate of 3 ml/min. During both operations, a metal catheter was inserted into the vena portae to collect the venous effluent. As soon as proper flow was apparent, rats were euthanized by perforation of the diaphragm. To allow for equilibration of the system, the intestine/colon was perfused for 25 min before initiation of the experimental protocol.

Each protocol started with a baseline period followed by the addition of the test substance applied either through the luminal tube or the vascular route. The venous effluent was collected for 1 min periods using a fraction collector. Effluent samples were immediately placed on ice and stored at −20 °C until analysis. As an indicator of the health of the small/large intestine, perfusion pressure was monitored throughout the experiment. The procedure is described in more detail elsewhere [17, 18, 22].

### Perfusion buffer and test substances

The perfusion buffer consisted of a modified Krebs-Ringer bicarbonate buffer supplemented with 3.5 mM glucose, 0.1% (w/v) bovine serum albumin (cat. no. 1.12018.0500, Merck, Denmark), 5% (w/v) dextran T-70 (to balance oncotic pressure; Pharmacosmos, Denmark), 5 mM of each fumarate, pyruvate and glutamate (Sigma Aldrich, Brøndby, Denmark) and 10 μM 3-isobutyl-1-methylxanthine (IBMX, cat no. 5879, Sigma Aldrich). The pH was adjusted to ~7.4 prior to experiments.

All test substances were purchased from Sigma Aldrich (Brøndby, Denmark) unless otherwise stated. Test substances included the following compounds infused to reach the following final concentrations; 50 mM l-valine (cat. no. V0513), 10 µM nifedipine (cat. no. N7634), 250 µM diazoxide (cat no. D9035).

### In vitro studies

GLUTag cells (generously provided by Professor Drucker from the University of Toronto in Canada) were cultured at 37 °C in an environment with 5% CO2 in Dulbecco’s modified Eagle’s medium (DMEM) with 5.6 mM glucose, supplemented with 10% fetal bovine serum, 1% penicillin (10,000 U/ml) and streptomycin (10,000 μg/ml), as well as 200 mM Glutamax (Thermofisher, Denmark). The cells were subcultured at a 1:5 dilution when they reached 80–85% confluence. For this study, we used cells with passage numbers ranging from 31 to 34.

In secretion studies, GLUTag cells were plated in 24-well culture plates at a density of 250,000 cells per well. Once the cells reached 80% confluence, which typically occurred within 24 to 48 h, the cells were subjected to a 30-minute period of starvation using freshly prepared Krebs-Ringer-HEPES (KRH) buffer. This KRH buffer contained 138 mM NaCl, 4.5 mM KCl, 4.2 mM NaHCO3, 1.2 mM NaH2PO4, 2.5 mM CaCl2, 1.2 mM MgCl2, and 10 mM HEPES, with a pH of 7.4, and it did not contain glucose or albumin. Following the starvation period, the cells were exposed to either KRH with 5.6 mM glucose (baseline secretion) or the same buffer containing varying concentrations of l-valine. We collected supernatants and processed them by centrifuging at 1500 × *g*, 4 °C, for 5 min to remove any debris. Supernatants were rapidly frozen and stored at −20 °C until further analysis. Experiments were conducted on three separate occasions, with each experiment having three technical replicates.

For calcium fluorimetry, we utilized a method employing Fluo-4 AM (Thermofisher). Cells were plated in a 96-well plate at a density of 50,000 cells per well and were cultured for 24–48 h. Before the experiment, the cells were loaded with 0.2% Fluo-4 AM, which was dissolved in HEPES-buffered Hank’s Balanced Salt Solution (HBSS, Thermofisher) containing 1 mM CaCl2, 1 mM MgCl2, and 0.5% probenecid (Thermofisher) for one hour. After a double-wash step with the same HBSS buffer without Fluo-4 AM, the plate was transferred to a FlexStation 3 from Molecular Devices. Fluorescence measurement was performed at excitation/emission λ of 494/506 nm. The background signal was registered for 20 s, followed by the addition of l-valine, using the instrument’s built-in automated pipetting system. Kinetic changes in intracellular calcium mobilization were registered for the total of 90 s. In some experiments, we introduced blockers to the wells after loading with Fluo-4 AM and waited for 15 min before taking readings. These blockers included nifedipine (2 mg/mL in DMSO), diazoxide (10 mg/mL in DMSO), and EDTA (0.5 M in NaOH), all purchased from VWR.

### Biochemical measurements

Peptide hormones were measured using in-house radioimmunoassays: total GLP-1 (the sum of 7-36NH_2_, 9-36NH_2_ and potential mid-terminal cleaved fragments) was measured using a C-terminal specific antibody targeting amidated forms of GLP-1 (code no. 89390) [23]. Total PYY (PYY_1–36_ + PYY_3–36_) was measured with a porcine antiserum (cat. no. T-4093; Bachem) [24]. For the in vitro studies, GLP-1 was measured using an antibody targeting the mid-region of GLP-1 (code no. 2135). Blood glucose was measured using a glucometer (AccuChek Mobile, cat. no. 05874149001; Roche Diagnostics, Mannheim, Germany). Plasma concentrations of active GLP-1 (7–36) were measured by sandwich ELISA (cat. no. 80-GLP1A-CH01, Alpco, Salem, New Hampshire). Total L-amino acid concentrations in venous effluents were quantified using a commercial colorimetric L-amino acid assay kit from Abcam (cat. no. ab65347).

### Calculations and statistical analysis

For analyses of GLP-1 secretion in perfusion experiments, hormone concentrations are presented in pM as mean values ± SEM. Mean concentrations were calculated for a 10-minutes baseline period before each stimulation/inhibition and for the last 10 min of each stimulation/inhibition period. For the baseline-subtracted concentrations, the individual baseline values were subtracted for each animal before calculating the mean value for the group. For within-group analyses, statistical significance was tested with a paired Student’s *t*-test or one-way ANOVA followed by Tukey’s post hoc analysis for multiple comparisons. For between-group analyses, statistical significance was tested with an unpaired Student’s *t*-test. The in vivo study was analyzed using two-way ANOVA followed by Šídák multiple comparisons test. Total areas under the curves were compared by one-way ANOVA followed by Tukey’s multiple comparison test. The in vitro studies were analyzed using one-way ANOVA. Post hoc analyses were performed using the Dunnett method.

Statistical testing and construction of graphs were done in GraphPad Prism version 10.1.2 (GraphPad Software, La Jolla, CA). *P* values < 0.05 were considered statistically significant.

## Discussion

As diets high in protein are theoretically advantageous for patients with T2D and obesity due to their satiating properties compared with carbohydrate- and lipid-rich meals [25–27], specific amino acids selected for their appetite-reducing and GLP-1 stimulating effect may represent an approach for nutritionally based modulation of GLP-1 secretion.

In this study, we investigated the effect of l-valine given orally to mice and compared the GLP-1 response with the response induced by oral glucose. In male mice, oral l-valine stimulated GLP-1 secretion similar to orally given glucose, demonstrating that l-valine is likewise a powerful stimulator of GLP-1 release in mice in vivo. However, the effect of l-valine on blood glucose regulation after an oral glucose tolerance test (OGTT) deserves further investigation, as l-valine could potentially help reduce blood glucose excursions after a carbohydrate-rich meal when given as a preload before the meal due to its GLP-1 stimulatory effect.

Furthermore, we investigated the intracellular mechanisms by which l-valine stimulates GLP-1 secretion. We found that co-administration of nifedipine, an inhibitor of V-gated Ca^2+^-channels, almost completely abolished the response to l-valine, indicating that l-valine induced GLP-1 secretion is dependent on activation of voltage-gated Ca^2+^-channels. The response was not completely inhibited, suggesting potential existence of other stimulatory pathways as well. l-valine also increased intracellular calcium levels in GLUTag cells.

We hypothesized that the membrane depolarization could be caused by a coupled transport of l-valine with positively charged Na^+^-ions into the GLP-1 secreting L-cells. Potential mediators of l-valine/Na^+^ absorption include the B^0^ transporters, which are expressed in the apical membrane of the small intestine and have a high affinity for l-valine [28]. When l-valine was infused in the presence of KCl as a replacement for NaCl in the intestinal lumen of the perfused rat small intestine, GLP-1 secretion was not affected, indicating that co-transport of l-valine and Na^+^ is not important for the depolarization necessary to activate the voltage-gated Ca^2+^-channels. In contrast, the effects of glucose on GLP-1 secretion, depending on co-transport with Na^+^ via sodium-glucose transporter 1 (SGLT1), is strongly inhibited by this maneuver [20]. However, other possible uptake mechanisms, such as l-valine coupled to H^+^, deserve further investigation. Though, there are important arguments against this idea. The proton-dependent amino acid symporter 1 (PAT1) is expressed in the apical membrane of the rat and human small intestine [29], however, l-valine does not seem to be a substrate for the transporter [30]. Even if substrate-specificity is different in the rat’s small intestine, another factor challenges the hypothesis as PAT1 is dependent on the Na^+^/H^+^ exchanger (NHE3) to create an H^+^-gradient for the transport. As the response to l-valine was not blocked in our study with a Na^+^ -free lumen (where the activity of NHE3 must be abolished), it contradicts the role of PAT1 in l-valine induced GLP-1 secretion.

We next investigated whether intracellular oxidation of l-valine followed by closure of K_ATP_-channels might be causing the depolarization. This mechanism has also been proposed for glucose/fructose-mediated GLP-1 secretion in-vitro and in the perfused rat small intestine [20, 31]. We found a pronounced inhibitory effect when opening K_ATP_-channels while stimulating with l-valine in the perfused rat small intestine, suggesting that changes in the membrane potential of GLP-1 secreting L-cells may play a crucial role in l-valine induced GLP-1 secretion. In GLUTag cells, the K_ATP_-channel opener reduced the effect of l-valine on intracellular calcium mobilization.

A limitation with the use of GLUTag cells, as well as other models for epithelial cells, is that the cells have lost their polarization, which may affect the amino acid sensing mechanisms, as different amino acid transporters are situated on the apical compared to the basolateral membrane [28]. l-valine could therefore potentially have entered GLUTag cells by mechanisms normally associated with basolateral uptake, which may affect the stimulatory pathway.

Another limitation in the perfused rat small intestine is that the second l-valine induced GLP‐1 response was lower than the first in all experiments. The relatively short washing period (20 min) between the two stimulations could explain this, as molecular sensors involved may have been saturated or desensitized by the first stimulation by the time of the second stimulation. Another limitation that needs to be considered is the applied concentration of l-valine (50 mM) in the perfused rat small intestine. This concentration is higher than that achieved after a regular meal, where the total amino acid concentration may reach around 50 mM [32–34]. Finally, as we only used male Wistar rats and male C57Bl/6JRj mice, we cannot exclude that l-valine works differently in female mice and rats (or other strains).

In the perfused rat colon, l-valine stimulated GLP-1 secretion as well as secretion of the hormone PYY, produced and released in parallel with GLP-1 from the L-cells. The hormone responses, however, were not as pronounced as seen in the perfused rat small intestine, suggesting that l-valine is not a powerful stimulator of gut hormone release from the distal part of the intestine. In line with this, the absorption of l-valine was also modest, supporting the theory that l-valine needs to be absorbed and metabolized to stimulate GLP-1 secretion. These observations are in agreement with the notion that nutrients are primarily absorbed in the proximal part of the small intestine [35]. Production of metabolites from amino acids, however, could play an important role in GLP-1 secretion from the colon, as the microbiota can ferment nutrients reaching the distal intestine (mostly colon) and produce metabolites, which may stimulate GLP-1 and PYY secretion [36–38]. The choice of using a total L-amino acid kit to measure the l-valine absorption was based on previous recovery testing of different commercial amino acid kits (unpublished data). The recovery of l-valine using the total L-amino acid kit (used in this study) is published elsewhere [18].

In this study, we demonstrated a potent effect of l-valine on GLP-1 secretion in both mice and rats. In a study by Elovaris et al., intraduodenal administration of l-valine at moderate and at supraphysiological doses to healthy young men had no effect on energy intake or blood glucose regulation, suggesting that l-valine does not have important glucoregulatory or appetite-suppressant effects in humans [39]. GLP-1 levels were not evaluated in that study, and the effects of a (DPP)-4 inhibitor were not investigated. In addition, it should be noted that the study design did not include a carbohydrate-containing meal or oral administration of l-valine, and the effects of l-valine on postprandial blood glucose were thus not evaluated. Further investigations are therefore needed to clarify the potency of l-valine (and other amino acids) in stimulating GLP-1 release in humans and to investigate the postprandial effect of l-valine on blood glucose regulation when given as a preload before a carbohydrate-containing meal.

Several amino acids have been demonstrated to stimulate GLP-1 release in vivo and in vitro, however, there are discrepancies between the reported stimulatory capacities of individual amino acids [40–46]. This may be due to the use of different experimental models, species differences and the use of different doses of amino acids. As future amino acid-based treatments seem to depend on identifying potent ligands to promote endogenous GLP-1 secretion in humans, and as the effects of oral amino acid supplementation on GLP-1 secretion in humans have been variable [44, 47–49], the effect of the individual amino acids on GLP-1 secretion in humans warrants further clarification.

**Fig. 5:**
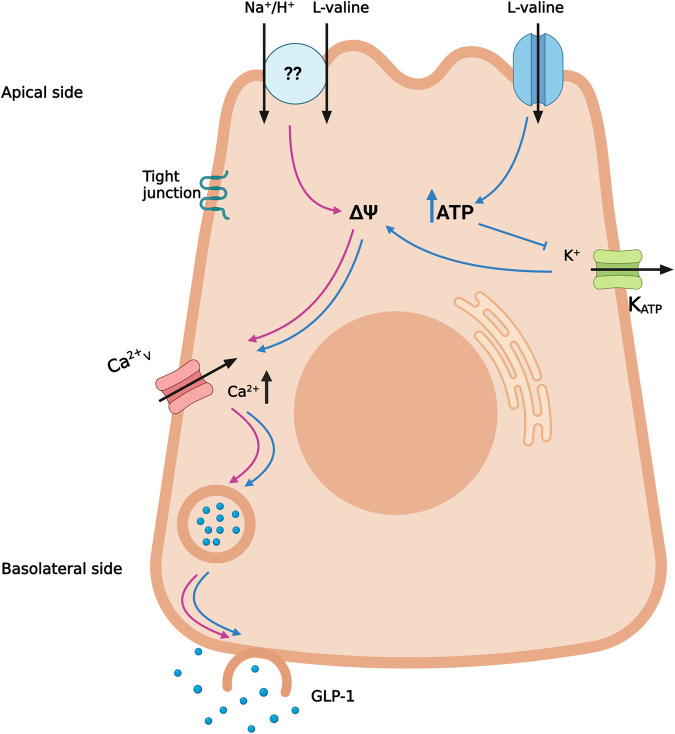
Proposed sensing mechanisms of l-valine by enteroendocrine L-cells. l-valine stimulates GLP-1 secretion through membrane depolarization and opening of V-gated Ca^2+^-channels. Membrane depolarization arises from intracellular metabolism of l-valine followed by closure of K_ATP_-channels. Coupled l-valine uptake with Na^+^ ions does not seem to be crucial for l-valine stimulated GLP-1 secretion. Whether coupled l-valine uptake with H^+^ contributes to promote membrane depolarization deserves further investigation. The figure is created with Biorender.com.

In conclusion, we found that l-valine is a powerful stimulator of GLP-1 secretion both in rats and mice (ex vivo and in vivo), and we suggest a mechanism for l-valine stimulated GLP-1 release involving intracellular metabolism of the amino acid, leading to the closure of K_ATP_-channels, membrane depolarization and activation of V-gated Ca^2+^-channels (Fig. 5).

## Data Availability

The datasets analyzed during the current study are available from the corresponding author on reasonable request.
